# Method Development and Validation of Montelukast in Human Plasma by HPLC Coupled with ESI-MS/MS: Application to a Bioequivalence Study

**DOI:** 10.3797/scipharm.1002-07

**Published:** 2010-06-04

**Authors:** Balasekhara Reddy Challa, Bahlul Z. Awen, Babu Rao Chandu, Mukkanti Khagga, Chandrasekhar Bannoth Kotthapalli

**Affiliations:** 1 Jawaharlal Nehru Technological University, Anantapur, Andhra Pradesh, 515002, India; 2 Siddharth Institute of Pharmacy, Nalanda Educational Society, Kantepudi, Sattenapalli, Guntur, 522438, India; 3 Faculty of Pharmacy, Al-Jabal Al-Gharbi University, Libya; 4 Jawaharlal Nehru Technological University, Hyderabad, 500072, India

**Keywords:** Montelukast, LC-ESI-MS/MS, Bioequivalence study

## Abstract

A simple, sensitive, and specific LC-ESI–MS/MS method for quantification of Montelukast (MO) in human plasma using Montelukast-*d*_6_ (MOD6) as an internal standard (IS) is discussed here. Chromatographic separation was performed on YMC-pack pro C_18_, 50 x 4.6 mm, S-3 μm column with an isocratic mobile phase composed of 10mM ammonium formate (pH 4.0):acetonitrile (20:80 v/v), at a flow-rate of 0.8 mL min^−1^. MO and MOD6 were detected with proton adducts at m/z 586.2→568.2 and 592.3→574.2 in multiple reaction monitoring (MRM) positive mode respectively. MO and MOD6 were extracted using acetonitrile as precipitating agent. The method was validated over a linear concentration range of 1.0–800.0 ng mL^−1^ with correlation coefficient (r^2^) ≥ 0.9996. The intraday precision and accuracy were within 1.91–7.10 and 98.32–99.17. The inter-day precision and accuracy were within 3.42–4.41% and 98.14–99.27% for MO. Both analytes were found to be stable throughout three freeze-thawing cycles, bench top, and autosampler stability studies. This method was utilized successfully for the analysis of plasma samples following oral administration of MO (5 mg) in 31 healthy Indian male human volunteers under fasting conditions.

## Introduction

**Fig. 1.**
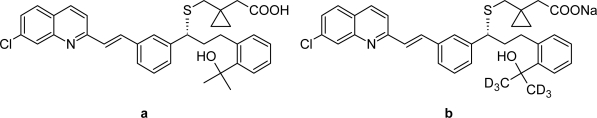
Chemical structures of Montelukast (a) and Montelukast-*d*_6_ sodium salt (b).

Montelukast, {1-[({(1*R*)-1-{3-[(*E*)-2-(7-chloroquinolin-2-yl)ethenyl]phenyl}-3-[2-(1-hydroxy-1-methylethyl)phenyl]propyl}sulfanyl)methyl]cyclopropyl}acetic acid, has a molecular formula of C_35_H_36_ClNO_3_S and a molecular weight of 586.18 g/mol (Fig. 1a).

Montelukast is a leukotriene receptor antagonist (LTRA) used for the treatment of asthma and to relieve symptoms of seasonal allergies. MO is usually administered orally. Mo is a CysLT_1_ antagonist; it blocks the action of leukotriene D4 on the cysteinyl leukotriene receptor CysLT_1_ in the lungs and bronchial tubes by binding to it. This reduces the bronchoconstriction otherwise caused by the leukotriene, and results in less inflammation. MO is more than 99% bound to plasma proteins with bioavailability of 63% to 73% and half life of 2.7–5.5 h and extensively metabolized by liver and excreted by biliary [1].

Several methods were developed for quantitative estimation of MO such as voltametric [10], capillary electrophoresis [12], spectroflurometry [15] spectrophotometry [17], and liquid chromatography (LC) [2–9, 11, 13, 14, 16]. Some methods were developed in pharmaceutical [11–14, 16, 17] and biological fluids [2–10, 15]. Moreover, voltametric [10], capillary electrophoresis [12], spectrophotometry [17], spectroflurometry [15] involves tedious procedure and too many steps which do not satisfy the determination of the samples. Quantification of MO in human plasma using HPLC was developed by few authors [3–8], which involves longer run time and are more expensive. Quantification of MO in human plasma using LC-MS/MS was developed by Bharathi D.V. et al. (2009) [2], where they observed a good linearity between the concentration ranges of 0.25–800.0 ng mL^−1^. However, the samples were pretreated with liquid–liquid extraction (LLE) and amlodipine was used as internal standard.

The proposed method involves high sensitivity, selectivity, and is reproducible for quantification of MO in plasma samples using samples acetonitrile precipitating agent by LC-ESI-MS/MS. Deuterated internal standard MOD6 (Fig. 1b) was used. We have developed and validated the method over a concentration range of 1.0–800.0 ng mL^−1^ using 200μL plasma samples. Limit of detection (LOD) was proved for 0.02 pg mL^−1^. Elution time was achieved in 2.8 min for both MO and MOD6. This method was developed and validated as per FDA guidelines and was successfully employed in the analysis of plasma samples following oral administration of MO (5 mg) in healthy human volunteers [18].

## Material and methods

### Standards and chemicals

MO was obtained from Varda Biotech Pvt. Ltd, Mumbai, India. MOD6 was obtained from TRC (Torrent research chemicals, Ontario) Canada. Acetonitrile, ammonium formate, formic acid were purchased from SD Fine Chemicals Ltd, Mumbai, India. Millipore water was used for all the experiments.

### Instrumentation

HPLC system (1200 series model, Agilent Technologies, Waldbronn, Germany), Mass spectrometry API 4000 triple quadrupole instrument (ABI-SCIEX, Toronto, Canada) using MRM.

### Detection

Turbo ionspray positive mode with unit resolution MRM was used for the detection. [M-H]^+^ (m/z 586.3) was monitored as the precursor ion for MO and fragmented at m/z: 568.2 was chosen as product ion. For internal standard, the [M-H]^+^ (m/z: 592.3) was monitored as the precursor ion and a fragmented at m/z 574.3 was monitored as the product ion. Mass parameters were optimized as source temperature 550°C, nebulizer gas 25 psi, heater gas 30 psi, curtain gas 20 psi, CAD gas 4 psi (nitrogen), ion spray voltage 5500 volts, source flow rate 800 μL min^−1^ without split, entrance potential 10V, declustering potential 65V for MO and 65V for MOD6, collision energy 25 V for both MO and MOD6, collision cell exit potential 8V for both MO and MOD6.

### Chromatographic conditions

YMC-pack pro C_18_, 50 x 4.6 mm, S-3 μm was selected as the analytical column. The mobile phase composition was 10mM ammonium formate (pH 4.0):acetonitrile (20:80 v/v). Flow rate of the mobile phase was set at 0.8 mL min^−1^ and 10 μL injection volume was used. Column temperature was set at 45°C. MOD6 was found to be appropriate internal standard. Retention time of MO and MOD6 were found to be 2.8 ± 0.2 min, with overall runtime of 5 min.

### Preparation of standards and quality control (QC) Samples

Standard stock solutions of MO (100 μg mL^−1^) and MOD6 (100 μg mL^−1^) were prepared in methanol. MOD6 standard solutions (400 ng mL^−1^) were prepared in 50% methanol from MOD6 standard stock solution. Standard stock solutions of MO were added to drug-free human plasma to obtain MO concentration levels of 1.00, 2.00, 5.00, 10.00, 50.00, 100.00, 200.00, 400.00, 600.00, and 800.00 ng mL^−1^ for analytical standards and 1.00, 3.00, 240.00, and 560.00 for quality control standards and stored in a −30°C set point freezer until analysis. Standard stock solutions and IS Standard solutions were stored in refrigerator conditions 2–8°C until analysis. Aqueous standard solutions were prepared in a mixture of 10mM ammonium formate pH 4.0:acetonitrile(1:9 v/v) and stored in refrigerator conditions 2–8°C until analysis.

### Sample preparation

50 μL of MOD6 standard solution (400 ng mL^−1^) was added into labeled microcentrifuged tubes and spiked 200 μL of plasma sample (respective concentration) into each tube and vortexed briefly. Plasma samples were cleaned with 750 μL of precipitating agent acetonitrile and vortexed briefly for about 5 min. Then the samples were centrifuged at 14000 g.force for approximately 10 min at ambient temperature. The supernatant from each sample was transferred into pre-labeled auto sampler vials for injection. Extraction was carried out under the absence of white light.

### Recovery

The extraction recoveries of MO and MOD6 from human plasma were determined by analyzing quality control samples. Recoveries at three concentrations (3.00, 240.00, and 560.00 ng mL^−1^) were determined by comparing peak areas obtained from the plasma sample and the standard solution spiked with the blank plasma residue.

### Selectivity

The response (peak area) was determined in blank plasma samples (six replicates from different plasma) and spiked LOQ was prepared from the same plasma. The peak area of blank samples should not be more than 20% of the mean peak area of LOQ of MO and not more than 5% of MOD6. The precision and mean accuracy of LOQ concentrations must be ≤ 20 and ± 20 % respectively. The signal to noise (S/N) for LOQ was found to be ≥ 5.

### Analytical curves

The analytical curves were constructed using values ranging from 1.00 to 800.00 ng mL^−1^ of MO in human plasma. Calibration curves were obtained by weighted 1 quadratic model with log transformed regression analysis (y = ax^2^ + bx + c). (x=MO concentration in plasma sample, y=Area ratio of MO and MOD6)The ratio of MO peak area to MOD6 peak area was plotted against the ratio of MO concentration to that of MOD6 concentration in ng mL^−1^.Calibration curve standard samples and quality control samples were prepared in replicates (n=6) for analysis. Accuracy and precision for the back calculated concentrations of the calibration points should be within ≤ 15 and ± 15% of their nominal values. However, for LLOQ, the precision and accuracy should be within ≤ 20 and ± 20%.

### Stability (freeze–thaw, auto sampler, bench top, long term)

Low quality control and high quality control samples (n=6) were retrieved from deep freezer after three freeze–thaw cycles according to the clinical protocols. Samples were frozen at −30°C in three cycles of 24, 36, and 48 h. In addition, the long-term stability of MO in quality control samples was also evaluated by analysis after 55 days of storage at −30°C. Autosampler stability was studied following 76 h-storage period in the autosampler tray. Bench top stability was studied for 27-h period. Stability samples were processed and extracted along with the freshly spiked calibration curve standards. The precision and accuracy for the stability samples must be within ≤ 15 and ± 15%, respectively, of their nominal concentrations.

### Analysis of patient samples

The bioanalytical method described above was used to determine MO concentrations in plasma following oral administration of healthy human volunteers. Each volunteer obtained written informed consent before participating in this study. Thirty-one healthy volunteers were chosen as subjects and administered 5 mg dose (one 5 mg tablet) by oral administration with 240 mL of drinking water. The reference product, Singulair tablets (Merck&CO) 5 mg and test product, MO tablets (test tablet) 5 mg were used. Study protocol was approved by IEC (Institutional Ethical committee) as per ICMR (Indian council of medical research) and the research followed the ethical standard formulated in the Helsinki declaration of 1964, revised in 2000. Blood samples were collected as pre-dose (0) h, 5 min prior to dosing followed by further samples at 0.5, 1.0, 1.333, 1.667, 2.0, 2.333, 2.667, 3.0, 3.333, 3.667, 4.0, 4.5, 5.0, 6.0, 8.0, 10.0, 13.0, 16.0, 20.0, and 24 h. After dosing, 5 mL blood was collected each time in vaccutainers containing K_2_EDTA. A total of 42 (21 time points for test and 21 time points for reference) time points were collected from each volunteer. The samples were centrifuged at 3200 rpm, 10°C, 10 min, and stored at −30°C until sample analysis. Test and reference were administered to same human volunteers under fasting conditions separately with proper washing periods (40 days gap between test and reference doses) as per protocol approved by IEC.

### Pharmacokinetics and statistical analysis

Pharmacokinetics parameters from the human plasma samples were calculated by a noncompartmental statistics model using WinNon-Lin5.0 software (Pharsight, USA). Blood samples were taken for a period of 3 to 5 times the terminal elimination half-life (t_1/2_) and it was considered as the area under the concentration time curve (AUC) ratio higher than 80% as per FDA guidelines [19, 20]. Plasma MO concentration-time profiles were visually inspected and C_max_ and T_max_ values were determined. The AUC_0–t_ was obtained by trapezoidal method. AUC_0–∞_ was calculated up to the last measureable concentration and extrapolations were obtained using the last measureable concentration and the terminal elimination rate constant (K_e_). The terminal elimination rate constant (K_e_), was estimated from the slope of the terminal exponential phase of the plasma of MO concentration–time curve by means of the linear regression method. The terminal elimination half-life, t_1/2_, was then calculated as 0.693/K_e_. Regarding AUC_0–t_ and C_max_ bioequivalence was assessed by means of analysis of variance (ANOVA) and calculating the standard 90% confidence intervals (90% CIs) of the ratios test/reference (logarithmically transformed data). The bioequivalence was considered when the ratio of averages of log-transformed data was within 80–125% for AUC_0–t_ and C_max_.

## Results and Discussion

### Method development and validation

**Fig. 2.**
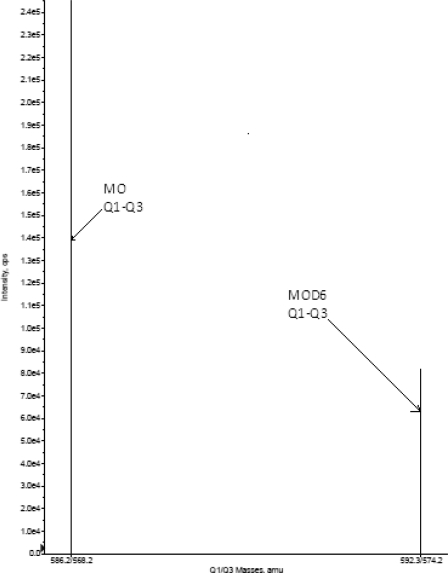
CAD mass spectra of MO Q1/Q3, MOD6 Q1/Q3.

LC-MS/MS has been used as one of the most powerful analytical tool in clinical pharmacokinetics for its selectivity, sensitivity, and reproducibility. The aim of the present study is to develop and validate a simple, sensitive, and rapid assay method for the quantitative determination of MO from plasma samples. A simple protein precipitation was used for extraction of MO and MOD6 from the plasma samples. Chromatographic conditions, especially the composition and nature of the mobile phase, were optimized through several trials to achieve the best resolution and increase the signal of MO and MOD6. The MS optimization was performed by direct infusion of solutions of both MO and MOD6 into the ESI source of the mass spectrometer. The critical parameters in the ESI source included the needle (ESI) voltage, which was directly related to the charged droplet formation and to the amount of gaseous ions formed. Capillary voltage was related to the gaseous ion guidance to the inside of the MS and was the last barrier between the atmospheric pressure and the high vacuum of the mass spectrometer. Other parameters, such as the nebulizer and the desolvation gases were optimized to obtain a better spray shape, resulting in better ionization and droplet drying to form, in our case, the protonated ionic MO and MOD6 molecules (Fig. 1). A CAD product ion spectrum for MO and MOD6 yielded high-abundance fragment ions of *m*/*z* 568.2 and *m*/*z* 574.2 respectively (Fig. 2). After the MRM channels were tuned, the mobile phase was changed from an aqueous phase to a more organic phase with acid dopant. A good separation and elution were achieved using 10 mM ammonium formate (pH 4.0):acetonitrile (20:80 v/v) as the mobile phase, at a flow-rate of 0.8 mL min^−1^ and injection volume of 10 μL.

### Selectivity

The analysis of MO and MOD6 using MRM function was highly selective with no interfering compounds (Fig. 3a)(selectivity was performed by using six different plasma lots, here showing only one blank plasma). MRM Chromatograms obtained from plasma spiked with MO (1.0 ng mL^−1^) and MOD6 (400 ng mL^−1^) are shown in Fig. 3b. LOQ S/N was found >5.

### Matrix effect

Matrix effect was determined by comparing peak area ratio obtained from the standard solution spiked with blank plasma before extraction and after extraction, and the precision for matrix effect at low, medium, and high concentrations must be less than 15%. The precision for the Montelukast matrix effect at all concentrations were determined to be less than 15%.

### Linearity, precision, and accuracy of calibration standards

Calibration curves were plotted as the peak area ratio (MO/MOD6) versus (MO/MOD6) concentration. Calibration was found to be linear over the concentration range of 1.00–800.00 ng mL^−1^. The RSD’s were less than 5% and the accuracy ranged from 96.82 to 102.43%. The determination coefficients (r^2^) were greater than 0.9996 for all curves (Tab. 1). These results indicate the adequate reliability and reproducibility of this method within the analytical range

### Precision and accuracy of quality control standards

Precision and accuracy for this method was controlled by calculating the intra and inter-batch variations at three concentrations (3.00, 240.00, and 560.00 ng mL^−1^) of QC samples in six replicates. As shown in Table 2, the intra-batch RSD’s were less than 7.10%.The intra and inter-day precision was within 1.91 to 7.10 and 3.41 to 4.40% and the intra and inter-day accuracy within 98.32 to 99.35% and 98.12 to 99.24% for MO (Table 2).

### Recovery

The recovery following the sample preparation using precipitation method with acetonitrile was calculated by comparing the peak area ratios of MO in plasma samples with the peak area ratios of solvent samples and was estimated at control levels of MO. The recovery of MO determined at three different concentrations 3.00, 240.00, and 560.00 ng mL^−1^ was found to be 76.05, 69.67, and 57.33% respectively. The overall average recovery of MO and MOD6 were found to be 67.68 and 64.87%, respectively.

### Limit of Detection (LOD)

The limit of detection was determined using aqueous standard solution. For Montelukast, 10 μL of a 20.00 pg mL^−1^ aqueous standard solution was injected and proved 0.20 pg limit of detection(LOD) for the instrument.

### Stability (freeze-thaw, auto sampler, bench top, long term)

Quantification of MO in plasma subjected to three freeze-thaw (−30°C to room temperature) cycles showed the stability of the analyte. No significant degradation of the MO was observed even after 76-h storage period in the auto sampler tray and the final concentrations of MO was between 97.0 to 100.8% of the theoretical values. In addition, the long-term stability of MO in QC samples after 55 days of storage at −30°C was also evaluated. The concentrations ranged from 99.3 to 97.3% of the theoretical values. These results confirmed the stability of MO in human plasma for at least 55 days at −30°C. (Tab. 3).

### Application to biological samples

**Fig. 4.**
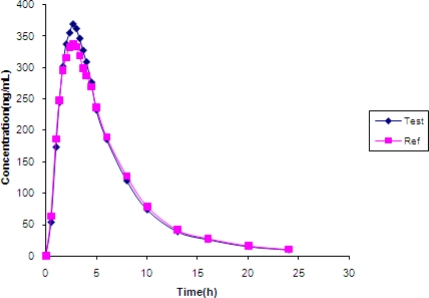
Mean plasma concentrations of test vs. reference after a 5 mg single oral dose (one 5 mg tablet) in 31 healthy volunteers.

The above-validated method was used in the determination of MO in plasma samples for establishing the bioequivalence of a single 5-mg dose (one 5 mg tablet) in 31 healthy human volunteers. Typical plasma concentration versus time profiles was shown in Fig. 4. All the plasma concentrations of MO were in the standard curve region and remained above the 1.00 ng mL^−1^ LOQ for the entire sampling period. The Pharmacokinetic parameters and 90%CI were shown in Table 4, 5. Therefore, it can be concluded that the two Montelukast formulations (reference and test) analyzed were bioequivalent according to regulatory requirements [19, 20] (Fig.4)

## Figures and Tables

**Fig. 3a.**
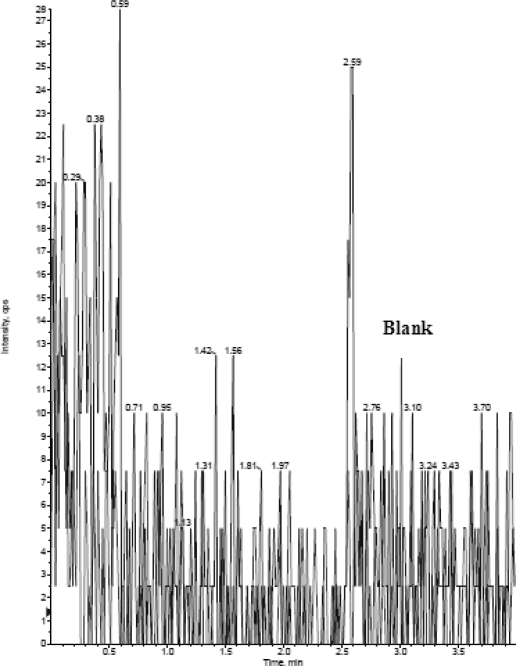
MRM chromatogram of MO and MOD6 in human blank plasma.

**Fig. 3b.**
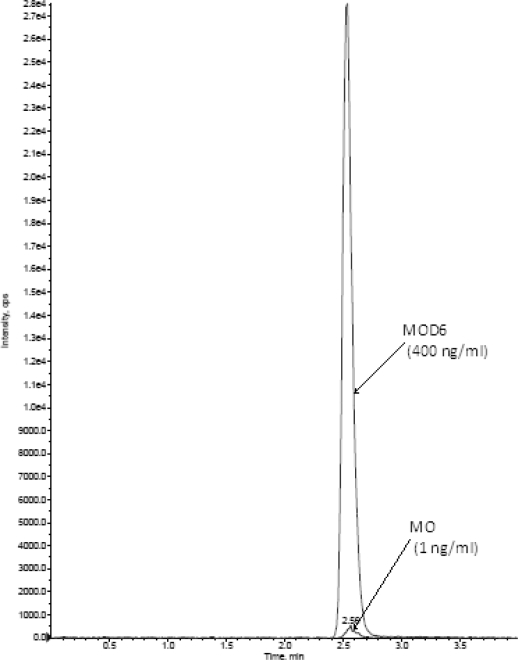
MRM chromatogram of MO and MOD6 in human plasma spiked with MO (1.00ng/ml), and MOD6 (400.00 ng/ml)[LOQ].

**Tab. 1.** Calibration curve details of the validation section

**Spiking Plasma Concentration (ng mL^−1^)**	**Concentration measured (ng mL^−1^) (mean)**	**RSD.^a^ (%) (*n* = 6)**	**Accuracy %**
1.00	1.00 ± 0.03	3.0	100.00
2.00	2.02 ± 0.08	4.0	101.00
5.00	5.05 ± 0.07	1.4	101.00
10.00	9.86 ± 0.18	1.8	98.63
50.00	48.42 ± 2.43	5.0	96.82
100.00	102.40 ± 4.22	4.1	102.43
200.00	204.07 ± 3.18	1.6	102.00
400.00	400.76 ± 8.46	2.1	100.21
600.00	605.05 ± 7.69	1.3	100.85
800.00	780.18 ± 7.71	1.0	97.52

a $\text{RSD}\cdot =\frac{\text{Standard}\;\text{deviation}}{\text{Mean}\;\text{concentration}\;\text{measured}}\cdot 100$

**Tab. 2.** Precision and accuracy (analysis with spiked plasma samples at three different concentrations)

**Spiked plasma concentration (ng mL^−1^)**	**Within-run**			**Between-run**		

	**Concentration measured (*n*=6) (ng mL^−1^)**	**RSD^a^ (%)**	**Accuracy %**	**Concentration measured (*n*=30) (ng mL^−1^)**	**RSD^a^ (%)**	**Accuracy %**
3.00	2.95 ± 0.21	7.10	98.32	2.95 ± 0.13	4.40	98.31
240.00	238.31 ± 4.60	1.91	99.35	238.13 ± 8.21	3.41	99.24
560.00	552.79 ±32.12	5.84	98.70	549.51 ± 20.79	3.81	98.12

a $\text{RSD}\cdot =\frac{\text{Standard}\;\text{deviation}}{\text{Mean}\;\text{concentration}\;\text{measured}}\cdot 100$

**Tab. 3.** Stability of Montelukast in human plasma

**Spiked plasma Concentration**	**Concentration found (ng/mL) mean ± SD**	**Precision (%CV) or RSD^a^**	**Accuracy (%)**
Room temperature stability for 27 h in plasma			

3.00	2.91 ± 0.08	2.79	97.00
560.00	543.00 ± 8.76	1.61	96.96

Auto sampler stability for 76 h			

3.00	2.91 ± 0.06	2.08	97.00
560.00	564.33 ± 13.71	2.43	100.77

Long-term stability for 55 days at −30°C			

3.00	2.98 ± 0.14	4.60	99.33
560.00	544.83 ± 11.02	2.02	97.29

Freeze and thaw stability at 48 h			

3.00	2.93 ± 0.08	2.69	97.67
560.00	541.83± 12.38	2.29	96.76

a $\text{RSD}\cdot =\frac{\text{Standard}\;\text{deviation}}{\text{Mean}\;\text{concentration}\;\text{measured}}\cdot 100$

**Tab. 4.** Mean pharmacokinetic parameters of Montelukast in 31 healthy human volunteers after oral administration of 5 mg of test and reference products

**Pharmacokinetic parameter**	**Reference**		**Test**	

	**Mean±SD**	**CV%**	**Mean±SD**	**CV%**
C_max_ (ng mL^−1^)	338.23 ± 126.44	37.38	369.29 ± 137.35	37.19
AUC_0–t_ (ng.hr/ml)	2416.53 ± 66.29	2.74	2417.26 ± 63.58	2.63
AUC_0–∞_ (ng.hr/ml)	2486.54 ± 72.15	2.90	2490.26 ± 71.43	2.87
t_max_ (hr)	2.67	–	2.67	–
t_1/2_	4.54	–	4.99	–

AUC_0–∞_ … Area under the curve extrapolated to infinity;

AUC_0–t_ … Area under the curve up to the last sampling time;

C_max_ … The maximum plasma concentration

T_max_ … The time to reach peak concentration

**Tab. 5.** 90% Confidence intervals for log-transformed pharmacokinetic parameters of Montelukast after administration of 5 mg of test and reference products in 31 healthy human volunteers

**Pharmacokinetic parameter**	**C_max_ (T/R)**	**AUC_0–t_ (T/R)**	**AUC**0–∞** (T/R)**
90% CI	109.19	100.03	100.15
